# Insight into the Metal–Support Interaction of Pt and *β*-MnO_2_ in CO Oxidation

**DOI:** 10.3390/molecules28196879

**Published:** 2023-09-29

**Authors:** Tiantian Zhang, Jiacheng Xu, Yan Sun, Shiyu Fang, Zuliang Wu, Erhao Gao, Jiali Zhu, Wei Wang, Shuiliang Yao, Jing Li

**Affiliations:** 1 School of Environmental Science and Engineering, Changzhou University, Changzhou 213164, Chinayansun.123@foxmail.com (Y.S.);; 2 School of Material Science and Engineering, Changzhou University, Changzhou 213164, China; 3 Key Laboratory of Advanced Plasma Catalysis Engineering for China Petrochemical Industry, Changzhou 213164, China

**Keywords:** metal–support interaction, CO oxidation, terminal-type oxygen, bridge-type oxygen, Pt, MnO_2_

## Abstract

Pt-based catalysts exhibit unique catalytic properties in many chemical reactions. In particular, metal–support interactions (MSI) greatly improve catalytic activity. However, the current MSI mechanism between platinum (Pt) and the support is not clear enough. In this paper, the interaction of 1 wt% Pt nanoparticles (NPs) on *β*-MnO_2_ in carbon monoxide (CO) oxidation was studied. The Pt on *β*-MnO_2_ inhibited CO oxidation below 210 °C but promoted it above 210 °C. A Pt/*β*-MnO_2_ catalyst contains more Pt^4+^ and less Pt^2+^. The results of operando DRIFTS-MS show that surface-terminal-type oxygen (M=O) plays an important role in CO oxidation. When the temperature was below 210 °C, Mn=O consumption on Pt/*β*-MnO_2_ was less than *β*-MnO_2_ due to Pt^4+^ inhibition on CO oxidation. When the temperature was above 210 °C, Pt^4+^ was reduced to Pt^2+^, and Mn=O consumption due to CO oxidation was greater than *β*-MnO_2_. The interaction of Pt and *β*-MnO_2_ is proposed.

## 1. Introduction

CO is a colorless, odorless, but toxic, gas [1]. CO is mainly emitted from vehicle combustion and industrial processes [2,3]. It is not only hazardous to human health, but also indirectly contributes to global warming [4]. Catalytic oxidation technology is a considerably effective method for reducing CO emission because it has high economic feasibility, lower cost, and lower secondary pollution generation [5]. Transition metal oxide catalysts have shown promising applications due to their low cost and good poisoning and sintering resistance [6]. MnO_2_ is widely used in catalytic oxidation reactions due to its variable valence states and good oxygen-storage and oxygen-release capacities [7,8,9]. MnO_2_ can form a variety of crystal structures, such as *α*-, *β*-, *γ*-, and *δ*-MnO_2_, according to [MnO_6_] as a backbone arrangement [7]. Among them, *β*-MnO_2_ has a thermodynamically stable phase and high crystallinity, which has become one of the current research hotspots [10]. It is still a challenge for MnO_2_ to perform CO oxidation at low temperatures [11,12].

Loading a noble metal onto a support is an effective way to improve catalytic activity. Noble metal–supported catalysts have been widely used in CO oxidation due to the MSI [2]. Among them, platinum (Pt)-based catalysts have attracted wide attention for their excellent catalytic activity [13]. Zhu et al. used a Pt/Al_2_O_3_ catalyst to convert formaldehyde at room temperature [14]. Huang et al. prepared a Pt/CeO_2_-TiO_2_ catalyst and found that it has high catalytic activity and selectivity for CO + NO reactions [15]. Cui et al. prepared an octahedral Fe_3_O_4_ to support Pt to remove formaldehyde and found that the catalyst has a high activity and stability [16]. Ru et al. synthesized Pt/(*α*-, *γ*-, and *δ*-) MnO_2_ catalysts for methanol oxidation, and showed that the temperatures for methanol oxidation on Pt/(*α*-, *γ*-, and *δ*-) MnO_2_ catalysts can be reduced in comparison with MnO_2_ [17].

MnO_2_ has two typical oxygen species: terminal-type oxygen (Mn=O) and bridge-type oxygen (Mn-O-Mn) [18]. Mn=O plays an important role in the CO oxidation process [19]. MSI can inhibit the growth of small noble metal nanoparticles and optimize the bonding structure and electron transfer, and, thus, improve catalytic performance [13]. Dispersion of Pt NPs onto MnO_2_ is an efficient method to weaken Mn=O and Mn-O-Mn bonds and generate oxygen vacancies through MSI between interfaces of Pt NPs and MnO_2_ [20]. However, some studies have shown that the strong interactions may limit catalytic reactions and weaken catalytic activity [13]. Miao et al. found that excessively strong MSI does not promote O_2_ adsorption [13,21]. At present, the interaction between Pt and the support is not clear enough. This study aims to gain insight into the interaction of Pt and MnO_2_ in CO oxidation.

Operando diffuse reflectance infrared Fourier transform spectroscopy (DRIFTS) can monitor surface species and oxygen vacancies under a real reaction condition and is often used to study catalytic reaction mechanisms [10]. Xu et al. used H_2_ as a probe molecule to study the role of oxygen species on the surface oxygen species of *β*-MnO_2_ of a low amount of M=O using operando DRIFTS coupled with mass spectrometry (MS) [10]. Li et al. studied the effects of acid gases (NO, HCl, and SO_2_) on mercury oxidation over CeO_2_-WO_3_/TiO_2_ catalysts through in situ DRIFTS and showed that NO_2_ and nitrate are active centers of mercury oxidation [22]. Using CO as a probe molecule and in situ DRIFTS, Jang, et al. quantified a part of the catalytic active sites on PdOx, further elucidating the surface reconstruction of Pt-Pd bimetallic particles during the reaction [23]. Zhang et al. confirmed that Rh atoms are dispersed on Al_2_O_3_ and TiO_2_ using Fourier transform infrared spectroscopy (FTIR) [24].

In this study, 1 wt% Pt NPs loaded on *β*-MnO_2_ prepared with deposition–precipitation is used to explore the metal–support interaction of Pt and *β*-MnO_2_ during CO oxidation. The physicochemical properties of *β*-MnO_2_ and Pt/MnO_2_ catalysts are characterized using an X-ray diffractometer (XRD), high-resolution transmission microscopy (HRTEM), X-ray photoelectron spectroscopy (XPS), and paramagnetic resonance spectroscopy (EPR). The role of oxygen species on the surfaces of *β*-MnO_2_ and Pt/*β*-MnO_2_ catalysts in CO oxidation is revealed using operando DRIFTS-MS. The interaction of Pt and *β*-MnO_2_ in CO oxidation is proposed.

## 2. Results and Discussion

### 2.1. Physical Phase Characterization

**Figure 1:**
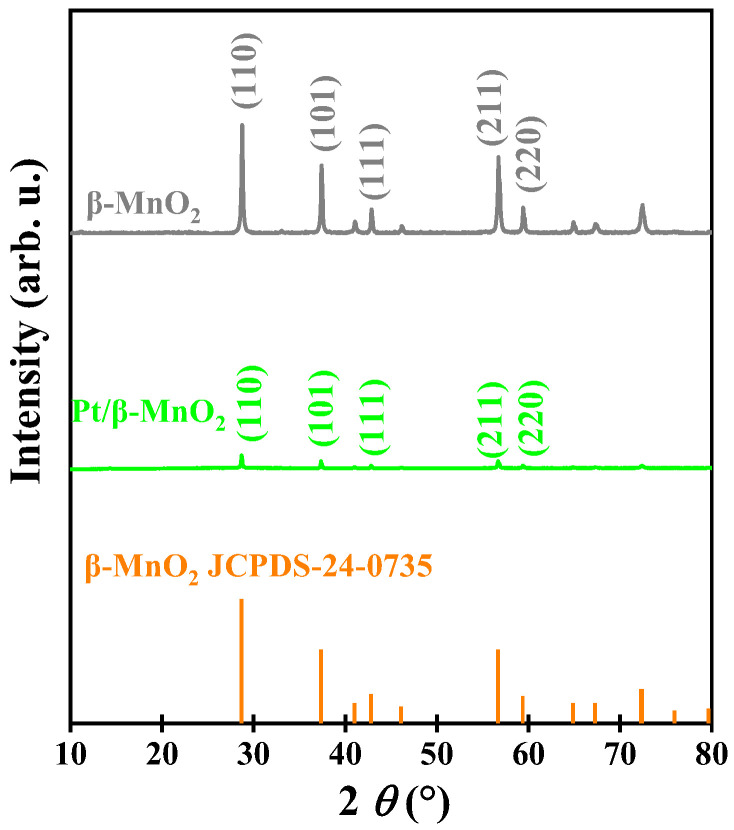
XRD patterns.

Figure 1 shows the XRD patterns of two catalysts. The diffraction peaks of *β*-MnO_2_ correspond to JCPDS 24-0735, and 2*θ* = 28.7°, 37.4°, 42.8°, 56.7°, and 59.5° correspond to (110), (101), (111), (211), and (220) crystal planes, respectively. *β*-MnO_2_ exhibits sharp and narrow diffraction peaks, suggesting that *β*-MnO_2_ has a high degree of crystallinity [25]. No diffraction peaks of Pt have been observed from XRD patterns, which may be due to small particle size, low Pt loading, or high dispersion of the Pt NPs [13,26,27,28,29]. The positions of the diffraction peaks of Pt/*β*-MnO_2_ are consistent with that of *β*-MnO_2_, indicating that the loading Pt did not change the crystal structure of *β*-MnO_2_. However, after loading Pt, the intensities of the diffraction peaks decreased, indicating that the crystallinity of *β*-MnO_2_ can be affected by Pt loading [30].

**Figure 2:**
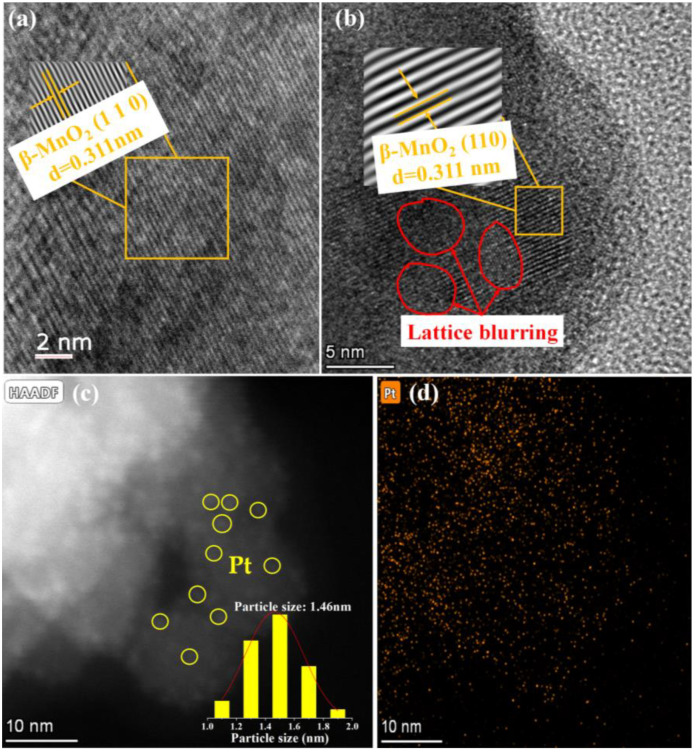
HRTEM images. (**a**) *β*-MnO_2_; (**b**) and (**c**) Pt/*β*-MnO_2_; (**d**) mapping of Pt on Pt/*β*-MnO_2_.

Figure 2 shows the HRTEM images of the two catalysts. The lattice spacing of *β*-MnO_2_ is 0.311 nm (Figure 2a), which is consistent with the (110) crystal face of *β*-MnO_2_. After loading Pt on *β*-MnO_2_, it was found that there are some crystal-plane blurs between the normal lattice stripes (inside the red circles in Figure 2b). This fact implied that the loading of Pt NPs caused defects in the catalyst [31]. Although no crystal planes of Pt species were observed in HRTEM, the presence of Pt NPs could be confirmed from HRTEM images (Figure 2c,d). The sizes of the Pt NPs were between 1.0 and 2.0 nm.

**Figure 3:**
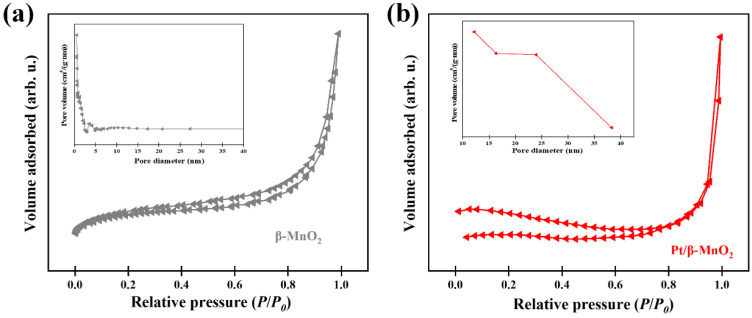
N_2_ adsorption/desorption isotherms and pore size distributions. (**a**) *β*-MnO_2_ and (**b**) Pt/*β*-MnO_2_.

The N_2_ adsorption isotherm and pore-size distribution curves of the two catalysts are shown in Figure 3. The physical and chemical properties of the two catalysts are summarized in Table 1. According to the IUPAC classification, the N_2_ adsorption isotherms of both catalysts are type IV isotherms. The specific surfaces before and after Pt loading are the same. However, the BJH pore-size distribution showed that *β*-MnO_2_ mainly has a microporous structure (<2 nm), while Pt/*β*-MnO_2_ is mainly of a mesoporous structure (2–50 nm). After loading Pt NPs, the pore volume of *β*-MnO_2_ decreased and the pore diameter increased, indicating that the small pores in *β*-MnO_2_ were blocked by Pt NPs.

### 2.2. Catalytic Activity

**Figure 4:**
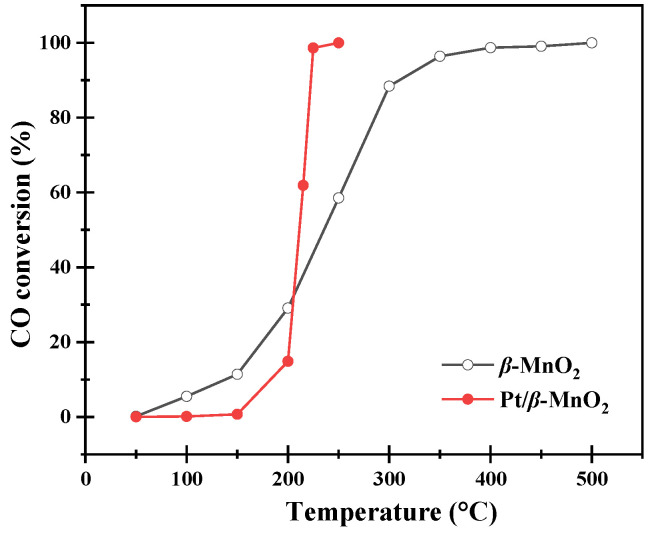
CO conversion at various temperatures.

The catalytic activities of the two catalysts are shown in Table 1 and Figure 4. Pt/*β*-MnO_2_ has *T*_50_ and *T*_90_ values, lower than *β*-MnO_2_. This means that loading Pt can enhance the activity of *β*-MnO_2_. The *TOF* value of Pt/*β*-MnO_2_ at 222 °C (*T*_90_) was 0.06 s^−1^; this *TOF* value is around the order of Pt NPs on SiO_2_ [32].

It should be noted that CO oxidation on Pt/*β*-MnO_2_ is lower than that of *β*-MnO_2_ at a temperature below 210 °C. This result suggested that Pt NPs inhibited CO oxidation below 210 °C by Pt NPs alone or in combination with *β*-MnO_2_. The inhibition mechanism is discussed later in Section 3.

### 2.3. Surface Chemical Structures

**Figure 5:**
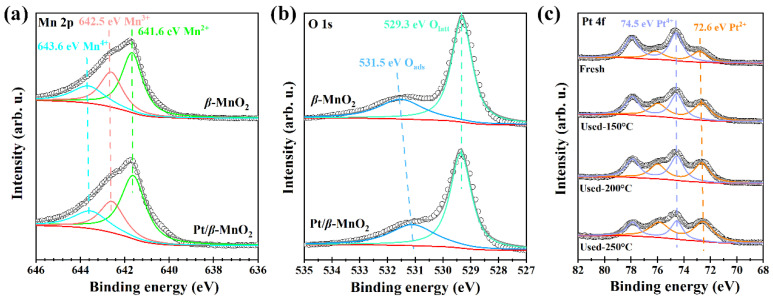
XPS spectra. (**a**) Mn 2p; (**b**) O 1s; and (**c**) Pt 4f.

The surface chemical structures of the two catalysts were characterized using XPS and Raman. Figure 5 shows the XPS spectra of the two catalysts. Each element is qualitatively identified and semiquantitatively calculated using the peak areas of each element [28,29]. The results are listed in Table 2. The peaks of 641.6, 642.5, and 643.6 eV (Figure 5a) correspond to Mn^2+^, Mn^3+^, and Mn^4+^, respectively [10]. The peak-area ratios of Mn cations with different valence states are shown in Table 2. Before and after Pt loading, the (Mn^2+^ + Mn^3+^)/Mn^4+^ ratio increased from 3.55 to 4.00, proving that Pt NPs on *β*-MnO_2_ can promote the formation of Mn^2+^ and Mn^3+^; those contribute to the formation of oxygen vacancies [33]. Moreover, the increase in the Mn^2+^ and Mn^3+^ ratio is also related to the formation of Pt-O-Mn [20].

The XPS spectra of O1s are shown in Figure 5b. The peaks at 529.3 and 531.5 eV correspond to lattice oxygen (O_latt_) and surface-adsorbed oxygen (O_ads_), respectively [10]. The peak-area ratios of the surface oxygen species on the two catalysts are listed in Table 2. The O_ads_/(O_ads_ + O_latt_) ratios of *β*-MnO_2_ and Pt/*β*-MnO_2_ are 0.30 and 0.33, respectively. Pt/*β*-MnO_2_ has more adsorbed oxygen than *β*-MnO_2_, which supports the fact that a higher (Mn^2+^ + Mn^3+^)/Mn^4+^ ratio can generate more oxygen vacancies on Pt/*β*-MnO_2_. The more surface-adsorbed oxygen is essential for catalytic oxidation [13]. Our finding also confirmed that catalysts containing more surface-adsorbed oxygen have better catalytic activity (Figure 4a). The XPS spectra of Pt 4f have broad peaks in the Pt 4f region (Figure 5c), indicating that Pt has different valence states. The peaks of 72.6 and 74.5 eV correspond to Pt^2+^ and Pt^4+^, respectively [13,20]. The peak-area ratios of different valence states before and after CO oxidation are given in Table 2. The Pt/*β*-MnO_2_ catalyst before CO oxidation mainly has Pt^+4^ and Pt^+2^, among which the Pt^4+^ ratio is as high as 0.68, showing that Pt^4+^ is the mainstay. The emergence of Pt^2+^ can be explained by the generation of Pt-O-Mn bonds, meaning that there is an interaction between Pt and *β*-MnO_2_ [33]. After the reaction at 150, 200, and 250 °C, the ratio of Pt^2+^/Pt_total_ gradually increased to 0.64. At the same time, the ratio of Pt^4+^/Pt_total_ decreased to 0.36. This finding clearly explores that Pt^4+^ was continuously reduced to Pt^2+^ during CO oxidation. Since Pt^2+^ is associated with the formation of Pt-O-Mn, it is speculated that Pt-O-Mn can promote CO oxidation. Combined with the low activity of Pt/*β*-MnO_2_ below 210 °C, it is concluded that Pt/*β*-MnO_2_ containing more Pt^4+^ and less Pt-O-Mn is not in favor for CO oxidation.

**Figure 6:**
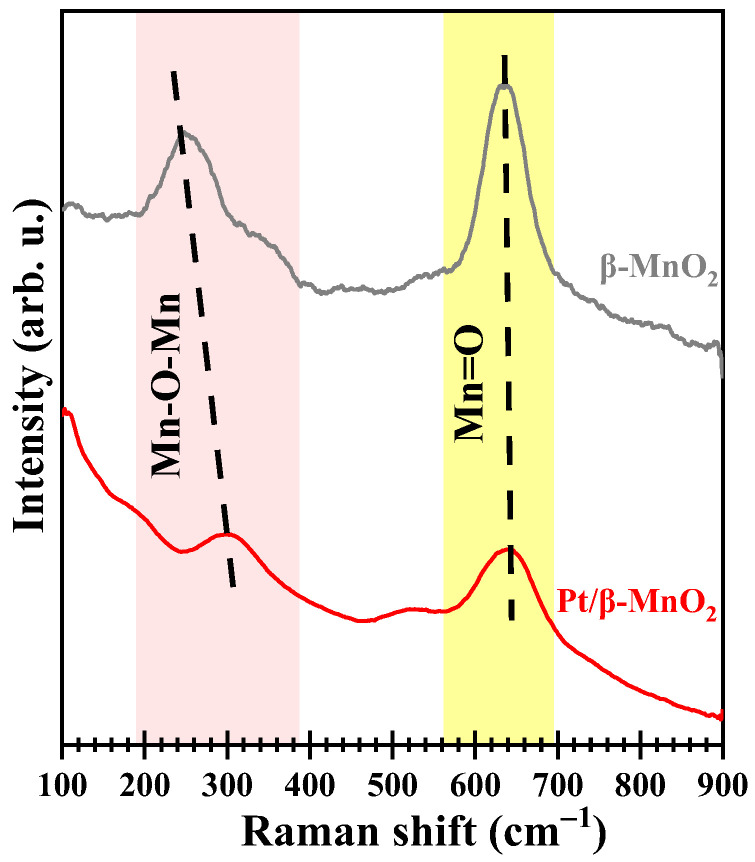
Raman profiles.

Figure 6 shows the Raman profiles of the two catalysts. The peak located in 500–700 cm^−1^ is attributed to the stretching of the [MnO_6_] octahedron [34]. The peak around 637 cm^−1^ is the symmetrical Mn=O tensile vibration perpendicular to the [MnO_6_] octahedral double strand. The low-intensity peak with a low wavenumber is attributed to the deformation mode of the metal–oxygen chain (Mn-O-Mn) in the MnO_2_ octahedral lattice [35]. Compared with *β*-MnO_2_, the vibration intensities of Mn=O and Mn-O-Mn after Pt loading have been weakened. This indicates that the mobility of lattice oxygen (Mn-O-Mn) increases after Pt loading [20]. The peak areas of Mn=O (A_Mn=O_) and Mn-O-Mn (A_Mn-O-Mn_) were calculated using LabSpec software. Pt/*β*-MnO_2_ has a higher A_Mn=O_/(A_Mn-O-Mn_ + A_Mn-O_) ratio than *β*-MnO_2_ (Table 2), implying that Pt/*β*-MnO_2_ has more adsorbed oxygen (Mn=O) than *β*-MnO_2_.

### 2.4. Reduction, Oxygen-Vacancy, and Electron-Transfer Analyses

**Figure 7:**
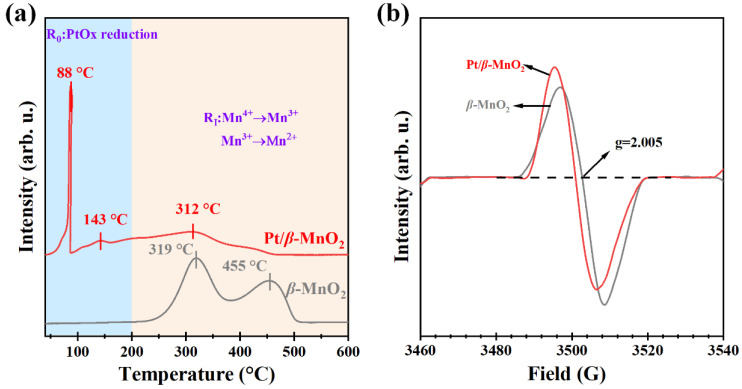
(**a**) H_2_-TPR profiles and (**b**) EPR spectra.

To further understand the interaction of Pt and *β*-MnO_2_, H_2_-TPR and EPR were performed. Figure 7a shows the H_2_-TPR profiles for two catalysts. The reduction temperature can be divided into two regions, *R*_0_ (50–200 °C) and *R_I_* (200–600 °C). Two reduction peaks (319 and 455 °C) were observed on *β*-MnO_2_, which were attributed to the reduction of Mn^4+^ to Mn^3+^ and then to Mn^2+^ [13,20]. After loading Pt, the *R*_0_ region has the reduction peak of PtOx at 88 °C and surface-adsorbed oxygen (O_ads_) at 143 °C [36]. Since the reduction peak in the *R*_0_ region is attributed to H_2_ spillover effects, which are caused by the formation of Pt-O-Mn bonds between *β*-MnO_2_ and adjacent Pt NPs [17,37], the Pt/*β*-MnO_2_ of peaks in the *R*_0_ region interacts between Pt and *β*-MnO_2_. Figure 7b shows the EPR spectra of the two catalysts. *β*-MnO_2_ has obvious electron paramagnetic resonance signal peaks at *g* = 2.005 [36], and the peak intensity can also reflect the oxygen-vacancy concentration. After loading Pt on *β*-MnO_2_, the signal at *g* = 2.005 is offset. This proves that there is electron transfer between Pt NPs and *β*-MnO_2_, which is consistent with typical MSI phenomena [38]. In addition, it is clear that the oxygen-vacancy concentration order is Pt/*β*-MnO_2_ > *β*-MnO_2_. Higher oxygen-vacancy concentrations correspond to better oxygen-mobility capacity, which is consistent with the characterization of H_2_-TPR. The oxygen vacancies of Pt/*β*-MnO_2_ increased significantly, mainly due to the fact that MSI affects the Mn=O bonds of the vector and forms more oxygen vacancies [36].

### 2.5. Surface Reactions Using Operando DRIFTS-MS

#### 2.5.1. Surface Reactions in CO Atmosphere

**Figure 8:**
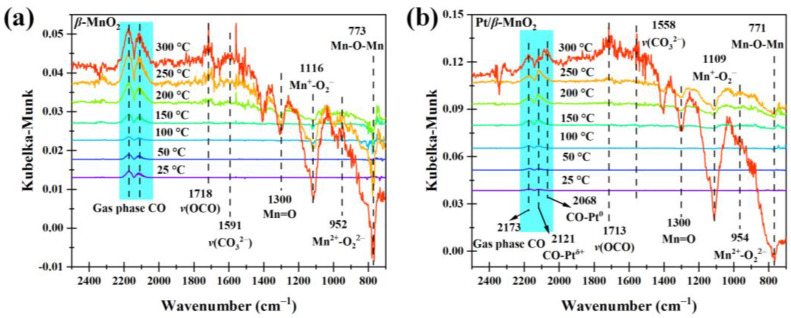
Operando DRIFTS spectra of two catalysts in 1% CO/Ar atmosphere. (**a**) *β*-MnO_2_ and (**b**) Pt/*β*-MnO_2_.

Figure 8 shows the operando DRIFTS spectra during CO oxidation on the two catalysts in a 1% CO/Ar atmosphere without O_2_. The peaks on *β*-MnO_2_ at 2173 and 2114 cm^−1^ belong to the gas-phase CO. On Pt/*β*-MnO_2_, the peak at 2173 cm^−1^ belongs to the gas-phase CO [19], the peak at 2121 cm^−1^ belongs to CO-Pt^δ+^ [39,40], and the peak at 2068 cm^−1^ is assigned to CO-Pt^0^ [39,40]. Peaks at 1300, 1112, 947, and 770 cm^−1^ are attributed to Mn=O, Mn^+^-O_2_^−^, Mn^+^-O_2_^2−^, and Mn^+^-O^2−^-Mn^+^, respectively [10,19]. The peak in 1605–1688 cm^−1^ is attributed to bidentate carbonate (*v*(OCO)), and the peak in 1463–1560 cm^−1^ belongs to monodentate carbonate (*v*(CO_3_^2−^)) [19].

**Figure 9:**
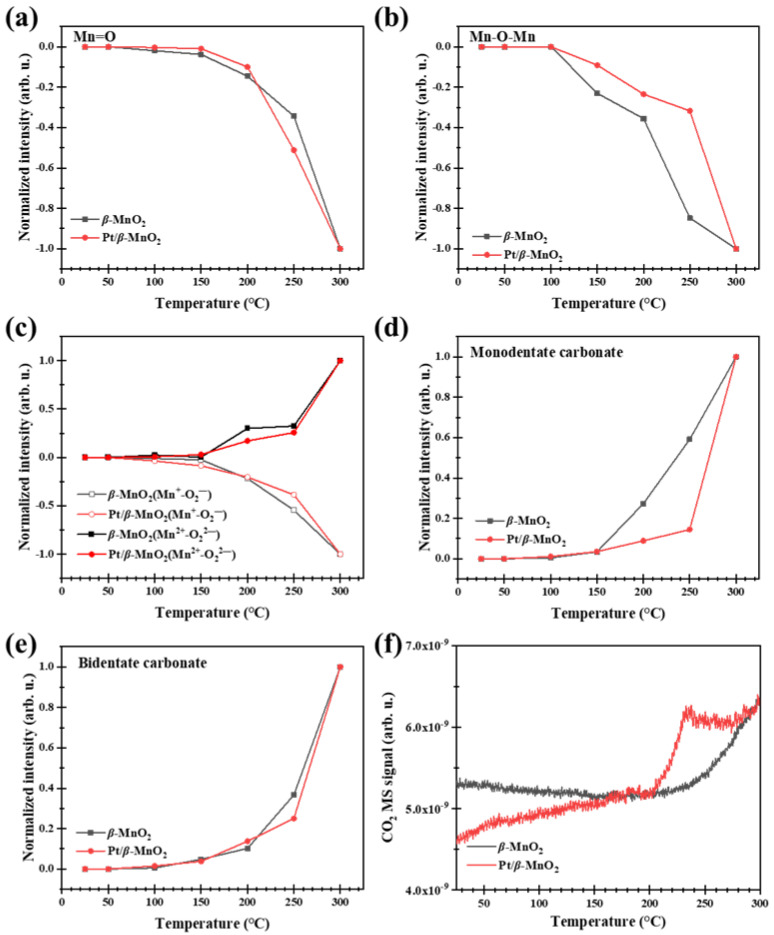
Normalized intensities of surface species on the surfaces of *β*-MnO_2_ and Pt/*β*-MnO_2_ catalysts (**a**–**e**) and MS signals (**f**) in a 1%CO/Ar atmosphere.

Figure 9 shows the normalized peak heights of the surface species and MS signals during CO oxidation on *β*-MnO_2_ and Pt/*β*-MnO_2_ in a CO atmosphere. As the temperature increased, the normalized intensities of Mn=O and Mn-O-Mn on *β*-MnO_2_ and Pt/*β*-MnO_2_ decreased (Figure 9a,b), indicating that oxygen species are continuously consumed due to their reactions with CO following Equations (1)–(4). The initial consumption temperature of Mn=O on *β*-MnO_2_ is 50 °C, obviously lower than that (150 °C) on Pt/*β*-MnO_2_, suggesting that Mn=O on *β*-MnO_2_ can react more easily with CO than that on Pt/*β*-MnO_2_. This fact also implied that Pt inhibited CO oxidation by the O in Mn=O. The initial consumption temperatures of Mn-O-Mn on *β*-MnO_2_ and Pt/*β*-MnO_2_ are higher than 100 °C, but the normalized peak height of Mn-O-Mn on *β*-MnO_2_ is less than that on Pt/*β*-MnO_2_ (Figure 9b), also suggesting that Mn-O-Mn on *β*-MnO_2_ can react easier with CO than that on Pt/*β*-MnO_2_; Pt inhibited CO oxidation by the O in Mn-O-Mn. The reactions of Mn=O and Mn-O-Mn with CO result in the formation of Mn=□ and Mn^2+^-□-Mn, where □ denotes an oxygen vacancy. As the initial adsorbed O_2_ in (O_2_^−^)Mn^2+^-O-Mn is reduced to (O_2_^2−^)Mn^2+^-□-Mn by CO (Equations (5) and (6)), the decrease in the normalized peak height of (O^2−^)Mn^2+^-O-Mn and increase in Mn^2+^-□-Mn above 50 °C (Figure 9c) proved the reactions in Equations (5) and (6). The CO oxidation products include surface carbonate and gaseous CO_2_. Figure 9d shows the normalized peak heights of monodentate carbonate (*v*(CO_3_^2−^)) on *β*-MnO_2_ and Pt/*β*-MnO_2_. The remarkable differences between *β*-MnO_2_ and Pt/*β*-MnO_2_ can be found within the temperature range of 100 and 300 °C, in which the normalized peak heights of monodentate carbonate on *β*-MnO_2_ are higher than that on Pt/*β*-MnO_2_, indicating that Pt inhibited CO oxidation to monodentate carbonate (Equation (7)). Figure 9e illustrates no obvious difference in the normalized peak heights of bidentate carbonate on *β*-MnO_2_ and Pt/*β*-MnO_2_; thus, Pt has no influence on bidentate carbonate formation following Equation (9) [41].

The CO_2_ MS signal from *β*-MnO_2_ is higher than that from Pt/*β*-MnO_2_ in the temperature range of 50–210 °C (Figure 9f), as Pt inhibited CO oxidation with O atoms in Mn=O and Mn-O-Mn.

Mn=O + CO → Mn=□ + CO_2_ (1)

Mn=O + CO-Pt^δ+^ → Mn=□ + CO_2_ (2)

Mn-O-Mn + CO → Mn-□-Mn + CO_2_ (3)

Mn-O-Mn + CO-Pt^δ+^ → Mn-□-Mn+CO_2_ (4)

(O_2_^−^)Mn-O-Mn + CO^+^ → (O_2_^2−^)Mn^2+^-□-Mn + CO_2_ (5)

(O_2_^−^)Mn-O-Mn + CO-Pt^δ+^ → (O_2_^2−^)Mn^2+^-□-Mn + Pt^δ+^ + CO_2_ (6)

Mn-O-Mn=O + CO → Mn-□-Mn-O-CO_2_ (7)

Mn=O + CO_2_ → Mn-O-CO_2_ (8)

Mn^2+^-O_2_^2−^+CO_2_ → Mn=O_2_-CO (9)

#### 2.5.2. Surface Reactions in CO/O_2_ Atmosphere

**Figure 10:**
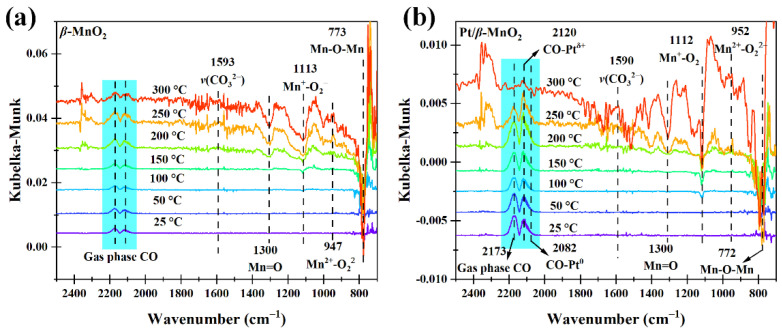
Operando DRIFTS spectra of two catalysts in a 1%CO/20% O_2_/Ar atmosphere. (**a**) *β*-MnO_2_ and (**b**) Pt/*β*-MnO_2_.

**Figure 11:**
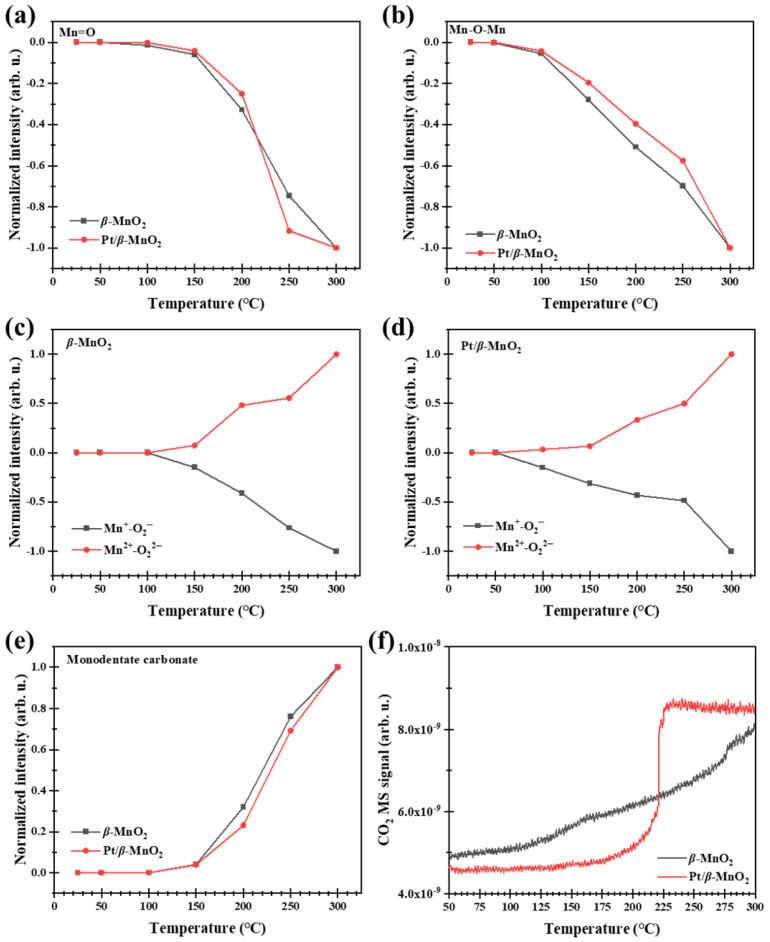
Normalized intensities of surface species on the surfaces of *β*-MnO_2_ and Pt/*β*-MnO_2_ catalysts (**a**–**e**) and MS signals (**f**) in a CO/O_2_/Ar atmosphere.

Figure 10 shows the DRIFTS-MS spectra of two catalysts during CO oxidation in a 1%CO/20%O_2_/Ar atmosphere. The normalized intensities of Mn=O and Mn-O-Mn obviously decreased above 150 and 100 °C, respectively (Figure 11a,b), which are similar to those (Figure 9a,b) during CO oxidation in the absence of O_2_. The normalized intensities of (O_2_^−^)Mn-O-Mn and Mn^2+^-□-Mn on Pt/*β*-MnO_2_ changed at 50 °C, lower than that (100 °C) on *β*-MnO_2_. Since the changes of (O_2_^−^)Mn-O-Mn and Mn^2+^-□-Mn are related to the formation of oxygen vacancy, Pt can promote the formation of oxygen vacancy in the presence of O_2_. The normalized intensities of monodentate carbonate *β*-MnO_2_ and Pt/*β*-MnO_2_ (Figure 11e) are similar to those (Figure 9e), explaining that O_2_ and Pt cannot influence the formation of monodentate carbonate.

The trend of the CO_2_ MS signal (Figure 11f) is basically similar to those during CO oxidation in the absence of O_2_, further proving that Pt can inhibit CO oxidation in the presence of O_2_ below 225 °C. This result is consistent with that in Figure 4a.

#### 2.5.3. Surface Reactions in O_2_ Atmosphere

**Figure 12:**
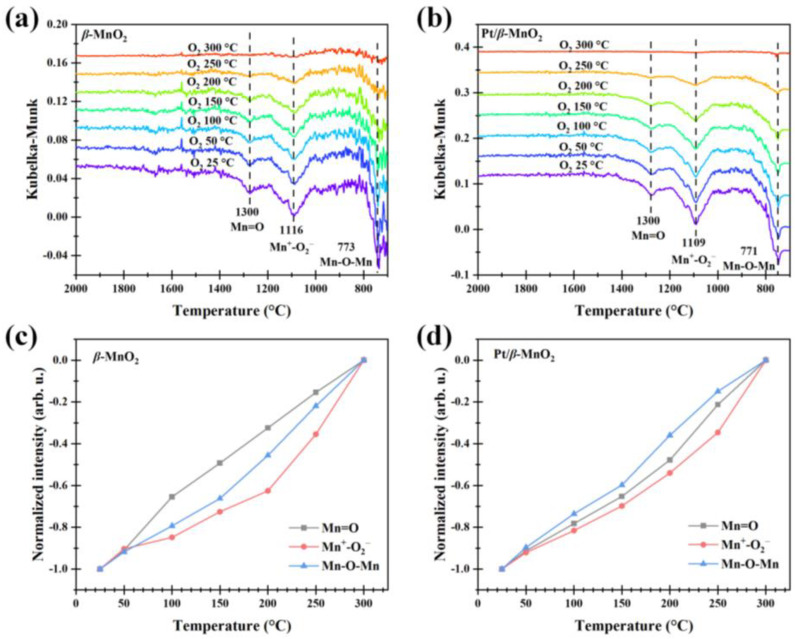
Operando DRIFT spectra of *β*-MnO_2_ (**a**) and Pt/*β*-MnO_2_ (**b**) in an O_2_ atmosphere. Normalized intensities of *β*-MnO_2_ (**c**) and Pt/*β*-MnO_2_ (**d**).

The normalized intensities of Mn=O and Mn-O-Mn after CO oxidation with or without O_2_ are all negative (Figure 9 and Figure 11), indicating that the regeneration rates of oxygen vacancies (Mn=□ and Mn-□-Mn) are lower than their consumption rates. To determine whether the oxygen vacancies can be regenerated, the catalysts with oxygen vacancies (generated via CO oxidation in a 1%CO/Ar atmosphere for 30 min at 300 °C) were heated from 25 °C to 300 °C with a ramp of 10 °C min^−1^ in a 20%O_2_/Ar atmosphere. The regeneration results are shown in Figure 12. Figure 12a,b show the DRIFTS spectra on *β*-MnO_2_ and Pt/*β*-MnO_2_ during regeneration. Figure 12c,d demonstrate that the normalized intensities of Mn=O and Mn-O-Mn increased to 0 at 300 °C, indicating that O_2_ decomposition at Mn=□ and Mn-□-Mn happened, resulting in the complete regeneration of Mn=O and Mn-O-Mn, where the O_2_ decomposition obeys Epling–Xu mechanism (Equation (10)) [42].

(O_2_^2−^)Mn-□-Mn → O=Mn-O-Mn (10)

## 3. Interaction of Pt and *β*-MnO_2_ in CO Oxidation

**Figure 13:**
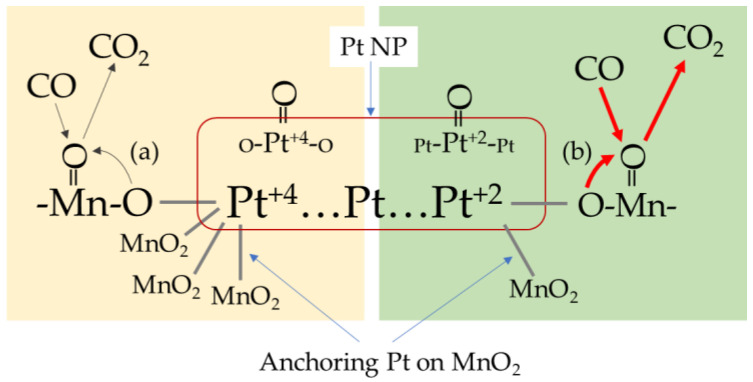
The interaction of Pt and *β*-MnO_2_ in CO oxidation.

Figure 13 shows the interaction of Pt NPs and *β*-MnO_2_ in CO oxidation. Pt NPs are anchored on MnO_2_ by Pt-O-Mn bonding, resulting in Pt^+4^ and Pt^+2^ valence states. CO is mainly adsorbed at Mn=O sites as the Pt loading is as low as 1 wt%. CO oxidation required additional O, which is from Mn=O or Pt-O-Mn and Mn-O-Mn. Step (a) indicates that O from Pt^+4^-O-Mn is difficult, causing the inhibition of CO oxidation. Step (b) is the easy process of O supplement from Pt^+2^-O-Mn to CO for its oxidation to CO_2_. At a temperature below 210 °C, the O in Pt^+4^-O-Mn cannot function as an O source, and Pt loading reduces the active sites of MnO_2_ for CO oxidation, as Pt is anchored at places having defects. Those are active sites for CO oxidation, and this results in the inhibition of Pt on CO oxidation. When the temperature is above 210 °C, the ratio of Pt^+4^ decreases, and the Pt^+2^ ratio increases, resulting in an increase in CO oxidation activity.

## 4. Materials and Methods

### 4.1. Catalyst Preparation

*β*-MnO_2_ was purchased from Aladdin, Shanghai, China. Chloroauric acid (H_2_Cl_6_Pt·xH_2_O) was from the Shanghai McLean Company (Shanghai, China).

The 1 wt% Pt/*β*-MnO_2_ was prepared using the deposition–precipitation method according to the method reported by Koo et al. [43]. Add H_2_Cl_6_Pt·xH_2_O (0.1 g mL^−1^), *β*-MnO_2_ (4 g), and distilled water (100 mL) to a beaker and mix thoroughly. Place the beaker in a 60 °C water bath and stir for 1 h; NH_3_·H_2_O was used to adjust the pH of the mixture to around 9 and then the mixture was stirred at 60 °C for 8 h. After stirring, the mixture was filtered and washed with hot distilled water and then dried at 60 °C for 12 h. After drying, the catalyst was put into a muffle furnace and calcined in an air atmosphere at 300 °C for 2 h.

### 4.2. Evaluation of Catalytic Activity

The CO oxidation activity of two catalysts (200 mg, 40–60 mesh) was measured in a fixed-bed flow reactor fed with 1% CO/20% O_2_ balanced with N_2_ at 100 mL min^−1^, with a gas-space velocity of 60,000 mL g^−1^ h^−1^ [27]. CO and CO_2_ concentrations were analyzed online using a gas chromatograph (GC9790II, Fuli, Taizhou, China) equipped with a flame ion detector with a catalyst convert to reduce CO and CO_2_ to CH_4_. Each catalyst was pretreated in 20% O_2_ with an N_2_ balance (80 mL min^−1^) atmosphere at 300 °C for 30 min. The CO conversion (x) and turnover frequency (*TOF*, s^−1^) were calculated using Equation (11) and Equation (12), respectively.

(11) $$x=\frac{{\left[CO\right]}_{in}-{\left[CO\right]}_{out}}{{\left[CO\right]}_{in}}\times 100$$

where [CO]_in_ and [CO]_out_ are the CO concentrations in gas streams from the inlet and outlet of the reactor, respectively.

(12) $$TOF=\frac{F\cdot {\left[CO\right]}_{in}\cdot x}{m_{cat}{\cdot m}_{Pt}/M_{Pt}}$$

where *F* is the total gas flow rate in mol s^−1^, *m_cat_* is the amount of catalyst in g, *m_Pt_* is the weight ratio of Pt in the catalyst, and *M_Pt_* is the molar atomic weight of Pt (195.08).

### 4.3. Catalyst Characterization

The operando DRIFTS-MS was used to monitor surface species and gaseous products during CO oxidation using the method reported elsewhere [10]. The catalyst characterization was performed as per [10].

## 5. Conclusions

In this study, 1 wt% Pt NPs loaded on *β*-MnO_2_ using deposition–precipitation was used to explore the effect of the metal–support interaction of Pt and *β*-MnO_2_ in CO oxidation. The main conclusions are as follows:

(1) After loading Pt on *β*-MnO_2_, it was found that Pt NPs (1–2 nm) were anchored on *β*-MnO_2_ and formed Pt-O-Mn interfaces. CO oxidation was inhibited at a temperature below 210 °C and promoted above 210 °C;
(2) The ratio of Pt^+2^ dominates CO oxidation. The ratios of Pt^+2^ before the reaction and after the reaction at 150, 200, and 250 °C were Pt/*β*-MnO_2_-fresh (0.32) < Pt/*β*-MnO_2_-used-150 °C (0.48) < Pt/*β*-MnO_2_-used-200 °C (0.51) < Pt/*β*-MnO_2_-used-250 °C (0.64). Pt^4+^ can convert to Pt^+2^ by heating;
(3) The Operando DRIFTS-MS results show that Mn=O plays an important role in CO oxidation. Below 210 °C, more Mn=O was consumed on *β*-MnO_2_ than on Pt/*β*-MnO_2_. When the temperature is higher than 210 °C, more Mn=O was consumed on Pt/*β*-MnO_2_ than on *β*-MnO_2_. It is speculated that the inhibition of activity below 200 °C after Pt loading is due to Pt^+4^, which causes less Mn=O consumption for CO oxidation;
(4) The mechanism of Pt and *β*-MnO_2_ interaction is proposed, where the Pt^+4^ and Pt^+2^ functions are clearly illustrated.

## Figures and Tables

**Table 1:** Specific surface areas, pore volumes, and pore diameters.

Catalyst	*β*-MnO_2_	Pt/*β*-MnO_2_
BET surface (m^2^ g^−1^)	1.29	1.29
Pore volume (cm^3^ g^−1^)	0.00354	0.00174
Pore diameter (nm)	5.83	32.74
*T*_10_ (°C)	140	182
*T*_50_ (°C)	235	212
*T*_90_ (°C)	310	222

**Table 2:** XPS and Raman analysis results.

Catalyst	XPS				Raman
	(Mn^2+^ + Mn^3+^)/Mn^4+^	O_ads_/O_total_	Pt^2+^/Pt_total_	Pt^4+^/Pt_total_	Mn=O Ratio *
*β*-MnO_2_	3.55	0.30			0.45
Pt/*β*-MnO_2_-fersh	4.00	0.33	0.32	0.68	0.66
Pt/*β*-MnO_2_-used-150 °C **			0.48	0.52	
Pt/*β*-MnO_2_-used-200 °C **			0.51	0.49	
Pt/*β*-MnO_2_-used-250 °C **			0.64	0.36	

* Mn=O ratio: M=O/(M=O + M-O-M). ** Pt/*β*-MnO_2_ catalysts after CO oxidation in 1% CO/20%/N_2_ atmosphere at 150 °C, 200 °C, and 250 °C for 1 h, respectively.

## Data Availability

The raw data are available from the corresponding author upon reasonable request.

## Sample Availability

Samples of the compounds are not available from the authors.
